# Effect of panretinal photocoagulation on the peripapillary retinal nerve fiber layer in diabetic retinopathy patients

**DOI:** 10.6061/clinics/2019/e1163

**Published:** 2019-11-11

**Authors:** Leandro Cabral Zacharias, Breno M.S. Azevedo, Rafael B. de Araujo, Marina R. Ciongoli, Marcelo Hatanaka, Rony C. Preti, Mario Luiz Ribeiro Monteiro

**Affiliations:** Divisao de Oftalmologia, Faculdade de Medicina FMUSP, Universidade de Sao Paulo, Sao Paulo, SP, BR

**Keywords:** Panretinal Photocoagulation, Peripapillary Retinal Nerve Fiber Layer, Scanning Laser Polarimetry, Diabetic Retinopathy, Optical Coherence Tomography

## Abstract

**OBJECTIVES:**

To determine the effect of panretinal photocoagulation (PRP) on the peripapillary retinal nerve fiber layer (RNFL) in nonglaucomatous patients with proliferative diabetic retinopathy (PDR).

**METHODS:**

This is a prospective, single center, observational study. Thirty-eight eyes of 26 diabetic patients underwent PRP for proliferative diabetic retinopathy. Peripapillary RNFL thickness was measured using scanning laser polarimetry (SLP) with variable corneal compensation (GDx VCC; by Carl Zeiss Meditec, Dublin, CA) and spectral-domain optical coherence tomography (OCT) (Heidelberg Spectralis, Carlsbad, USA) at baseline and 12 months after PRP was performed.

**RESULTS:**

Thirty-eight eyes of 26 patients (15 female) with a mean age of 53.7 years (range 26 to 74 years) were recruited. No significant difference was found among all RNFL thickness parameters tested by GDx VCC software (*p*=0.952, 0.464 and 0.541 for temporal-superior-nasal-inferior-temporal (TSNIT) average, superior average, inferior average, respectively). The nerve fiber indicator (NFI) had a nonsignificant increase (*p*=0.354). The OCT results showed that the average RNFL thickness (360° measurement) decreased nonsignificantly from 97.2 mm to 96.0 mm at 1 year post-PRP (*p*=0.469). There was no significant difference when separately analyzing all the peripapillary sectors (nasal superior, temporal superior, temporal, temporal inferior, nasal inferior and nasal thickness).

**CONCLUSION:**

Our results suggest that PRP, as performed in our study, does not cause significant changes in peripapillary RNFL in diabetic PDR patients after one year of follow-up.

## INTRODUCTION

Diabetic retinopathy (DR) is one of the most significant ocular complications related to diabetes mellitus (DM) and is one of the main causes of blindness (1). DM is also an important risk factor for chronic open angle glaucoma, and both diseases often coexist.

In cases of proliferative diabetic retinopathy (PDR), panretinal photocoagulation (PRP) is still considered the first-line treatment. Although PRP reduces the risk of severe vision loss (2), it has been shown that laser energy can cause destruction to all layers of the retina, including ganglion cells and the retinal nerve fiber layer (RNFL), and therefore generate visual field defects similar to that observed in glaucomatous damage (3). In such cases, visual field testing can be less helpful in evaluating glaucomatous damage.

With the development of clinically available imaging technology, previous studies showed that diabetic patients, with or without retinopathy, present thinner RNFL thickness than the normal population (4-6). Moreover, it has been suggested that ganglion cell loss secondary to PRP and ascending RNFL atrophy can change the optic disc appearance and topography (7).

Patients with glaucoma are traditionally managed by intraocular pressure (IOP) monitoring, assessing the structure of the optic disc and RNFL (through retinography and stereoscopic disc photographs), and functional evaluation using achromatic perimetry. With advancements in technology, serial measurements of RNFL thickness are routinely used to follow patients with glaucoma or suspected glaucoma because a decrease in RNFL thickness may indicate disease progression (8,9). Currently, quantitative and qualitative evaluation of RNFL can be performed by scanning laser polarimetry (SLP) and optical coherence tomography (OCT), aiding in the early detection of disease progression.

Previous studies have reported conflicting results of RNFL and optic disc topographic measurements after PRP treatment in diabetic patients (10-13). The current study investigated changes in RNFL thickness acquired using different image methods in diabetic patients after PRP.

## MATERIALS AND METHODS

This prospective observational cohort study enrolled patients from the retina service of the Ophthalmology Division of the University of Sao Paulo Medical School. The study protocol was approved by the local ethics committee and adhered to the tenets of the Declaration of Helsinki. Informed consent was obtained from all subjects.

The inclusion criteria for the study were as follows: diagnosis of PDR (due to type 1 or 2 DM), intraocular pressure <18 mmHg, nonglaucomatous optic disc characteristics at fundus examination, vertical cup-to-disc (C/D) ratio <0.7 and absence of media opacities. Subjects with a previous diagnosis or family history of glaucoma, any coexisting neuroophthalmic disease, uveitis, retinal vascular occlusion, optic disc neovascularization, diabetic macular edema (DME), corneal opacity or previous laser photocoagulation treatment were excluded from the study. Subjects with optic disk neovascularization were excluded due to the possible interference of the new vessels with the acquired images needed per protocol; patients with DME were excluded to avoid any interference of anti-VEGF intravitreal injections (indicated in most such cases) with the trial results.

All participants underwent complete ophthalmological examination at baseline, including best-corrected visual acuity (BCVA) with Snellen charts, Goldmann applanation tonometry, slit lamp biomicroscopy of anterior and fundus segments using a 78D lens (Volk, Mentor, OH, USA), and indirect binocular ophthalmoscopy. The RNFL of all patients was imaged by SLP and spectral-domain OCT at baseline and one year after the last laser treatment.

SLP is an imaging technology based on the principle that polarized light suffers a measurable shift (known as retardation) when it passes through the birefringent RNFL, and this shift is linearly related to the nerve fiber layer thickness (14). According to well-established methods, the peripapillary RNFL of all enrolled patients was assessed using SLP by GDx access in variable corneal compensator (VCC) mode (software version 5.5.1: Zeiss-Humphrey Systems, Dublin, California, USA). As previously described, GDx VCC automatically compensates for corneal birefringence (15). For consecutive exams, the software saves the acquisition position around the optic disk, which enables consecutive exams to be performed at the same anatomical location.

**Figure 1:**
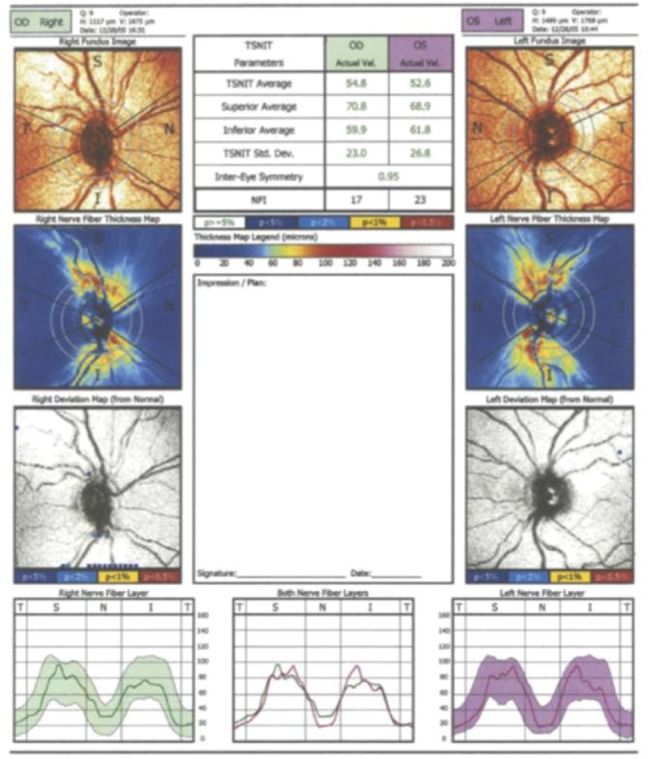
Printout of the retinal nerve fiber layer (RNFL) analysis with scanning laser polarimetry (SLP) with variable corneal compensation (GDx VCC).

In the current study, the following parameters contained at the standard printout of the GDx VCC instrument were analyzed: superior average, inferior average, TSNIT (360° RNFL thickness measured at the automatically defined 3.2-mm-diameter calculation circle: T, temporal sector; S, superior sector; N, nasal sector; I, inferior sector) average, TSNIT standard deviation, and the nerve fiber indicator (NFI). The NFI is calculated through an algorithm based on several RNFL measures and assigns a number from 0 to 100 to each eye. According to the manufacturer, higher NFI values correspond to a greater likelihood that the patient has glaucoma (16). Figure 1 is an example of a printout of the RNFL analysis obtained from a study eye using the GDx VCC software.

**Figure 2:**
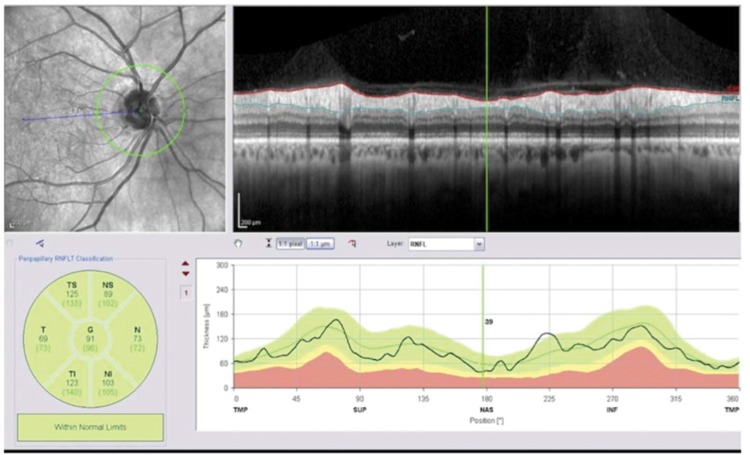
Example of the Spectralis spectral-domain OCT displaying the RNFL thickness parameters according to comparisons with a normative database population.

RNFL thickness was also measured using Spectralis spectral-domain optical coherence tomography (SD-OCT, software version 6.3.4; Heidelberg Engineering, Carlsbad, California, USA), according to protocols previously used in similar studies (17-19). A high-resolution protocol was used, and images were acquired by a single operator. The enrolled patients were asked to look at the internal fixation target, and a scan with a circle diameter of 3.45 mm was centered on the optic disc. To be accepted, images had to have a signal strength >15 dB, be well centered and have accurate segmentation. Follow-up mode was used to guarantee that the same position of the first scan circle was imaged in subsequent acquisitions. For this study, the following RNFL parameters, which are available in the standard software evaluation as shown in Figure 2, were evaluated: average RNFL, nasal superior, temporal superior, temporal, temporal inferior, nasal inferior and nasal thickness.

**Figure 3:**
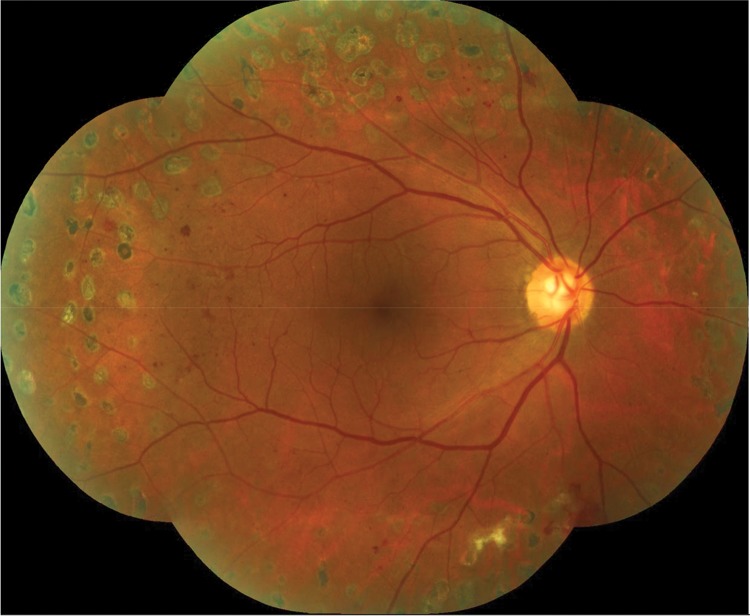
Retinography (montage) of the posterior pole and mid periphery exemplifying the pattern of laser photocoagulation performed in the study.

PRP treatment was carried out with a single spot green laser (Purepoint^®^ laser system, 532 nm wavelength, Alcon, Fort Worth, TX, USA). At least 1,500 peripheral laser photocoagulation burns were performed. The laser parameters used were a spot size of 250 μm, a pulse duration of 0.2 s and a sufficient power to cause moderate intensity burns following Early Treatment Diabetic Retinopathy Research Study (ETDRS) guidelines. PRP was carried out in 3 sessions, each session was 1 week apart. All patients were submitted to a complete ophthalmologic exam after 6 weeks to evaluate disease status and the need for further laser sessions. Figure 3 exemplifies the pattern of laser photocoagulation applied in the study and the distance between the laser burns and the optic disc margins.

Statistical analyses were performed using commercially available computer software (SPSS, ver. 23.0; SPSS, Chicago, USA). Normality was tested using the one-sample Kolmogorov-Smirnov test. The Wilcoxon signed ranks test was used to compare the RNFL parameters measured by SLP and OCT parameters before and after laser treatment. The statistical significance level was considered at *p*<0.05.

## RESULTS

This study included 42 eyes of 30 patients. Four eyes of 4 patients were excluded during the follow-up visits: one patient developed preretinal membranes causing tractional detachment and another developed vitreous hemorrhage in the eye; both of these patients were submitted to pars plana vitrectomy; another two eyes developed macular edema and were treated with intravitreal anti-VEGF (vascular endothelial growth factor) injections. Thirty-eight eyes of 26 individuals (15 female, 11 male) completed the one-year follow-up. The mean age was 53.7 years, ranging from 26 to 74 years.

No significant difference was found among all the RNFL thickness parameters tested by GDx VCC software (*p*=0.952, 0.464 and 0.541 for temporal-superior-nasal-inferior-temporal (TSNIT) average, superior average, inferior average, respectively). The NFI increased from 26.9±10.1 to 28.4±10.5, but the difference was not statistically significant (*p*=0.354). The data are summarized in Table 1.

The OCT results are presented in Table 2. The average RNFL thickness (360° measurement) decreased nonsignificantly from 97.2 μm to 96.0 μm at 1-year post-PRP (*p*=0.469). When all the peripapillary sectors were analyzed separately (nasal superior, temporal superior, temporal, temporal inferior, nasal inferior and nasal), an RNFL thickness reduction was also found but did not reach statistical significance (*p*=0.410, 0.413, 0.565, 0.168, 0.931 and 0.121, respectively).

## DISCUSSION

Laser photocoagulation decreases the risk of blindness in PDR patients, and despite the evidence of efficacy of antiangiogenic drugs, it is still considered the standard of care for the management of proliferative disease, according to The American Academy of Ophthalmology’s Preferred Practice Pattern for Diabetic Retinopathy (20), which valorizes the findings of the Diabetic Retinopathy Study (2) and the ETDRS (21) (level 1 evidence). While most visual complications in patients with PDR are related to retinal damage, it is not uncommon for patients to have glaucoma-associated visual loss either because of disease unrelated to diabetes or because of intraocular pressure elevation from PRP or treatment modalities such as corticosteroid injection. Therefore, while the causal relationship between DM and glaucoma in many cases remains unclear (22), the diagnosis of glaucoma in patients who have advanced DR can be challenging, particularly when submitted to PRP, as laser treatment can cause visual field changes that may mimic the field loss observed in glaucomatous patients.

Even in cases without DR, diabetes can cause alterations in the retinal structure. RNFL thickness measurements have shown that diabetic patients even without clinically evident retinopathy and those with nonproliferative disease have thinner nerve fiber layer thickness than controls without diabetes (4-6,23). This finding might be explained by the upregulation of enhanced apoptosis promoting factors causing early death of ganglion cells in diabetic patients (24).

In the current study, two different acquisition technologies were used to analyze the effects of PRP on RNFL. As both OCT and GDx showed no statistical change in RNFL thickness, we believe the use of different technologies reinforces our findings. Considering that the circular scan used for OCT acquisition is larger than the scan used on GDx (3.4 *versus* 3.2 mm), one would expect larger numerical OCT measurements for GDx than OCT. However, numerical data or maps from the two different technologies should not be directly compared, as GDx and OCT technologies are based on two different optical properties. While GDx uses changes in the polarization properties of light to determine thickness, OCT is based on the interference of light that passes through the tissue of the eye with a reference optical path to determine thickness at each point and construct an image. Therefore, these two technologies do not allow direct comparison, but there are numerous publications that demonstrate their importance in diagnosing glaucoma and monitoring RNFL thickness (4-6,8-11,16,17,19).

Some studies have shown conflicting evidence regarding the effect of PRP on RNFL peripapillary thickness. Ritenour et al. (11) observed an increase in RNFL thickness up to 6 months after laser photocoagulation in their study using SLP. Similar findings were reported by Maia OO et al. (10) using time-domain OCT. Using the same OCT technology, Lim et al. (12) compared healthy eyes with PDR eyes undergoing PRP and noticed that the group treated with laser presented a thinner RNFL thickness than the control group, although the authors did not compare pre- and post-laser RNFL thickness in the same group. On the other hand, using the same technology as in the above-mentioned studies, Kim and Cho (13) did not find a statistically significant decrease in the RNFL thickness of the group treated with PRP compared with the untreated control group. Lee et al. (25) performed one of the few prospective studies on this subject using time-domain OCT. They found that RNFL thickness tends to increase in the first 6 months and then decrease 2 years after PRP compared with pretreatment peripapillary RNFL thickness. Therefore, a short-term increase in RNFL thickness has been consistently reported in most studies (10,13,25), but the long-term effect of laser photocoagulation on RNFL remains controversial.

In this study, two different technologies, SLP and OCT-SD, were employed to measure the RNFL peripapillary thickness, and our results showed no statistically significant difference for either method between one year after conventional PRP and baseline before starting treatment. These results are similar to those found in another prospective trial that used laser scan pattern (PASCAL) photocoagulation, which is a new form of multispot laser treatment with shorter pulse duration (and consequently, is more restricted to retinal damage) than conventional single spot PRP (26). One hypothesis to explain the inconsistent findings in the literature related to RNFL changes after PRP is that different laser delivery may cause different degrees of inner retinal changes. While in our study a spot size of 250 µm was used, a larger spot size or laser intensity may be related to greater inner retinal damage and, therefore, more reduction in RNFL thickness due to retrograde axonal loss.

A recently published subanalysis of protocol S, a multicentric and randomized clinical trial that compared PRP or anti-VEGF for PDR cases, evaluated the effect of PRP and ranibizumab intravitreal injections on RNFL peripapillary thickness and found that after 2 years of follow-up, eyes treated with intravitreal anti-VEGF injections had greater RNFL thinning than eyes submitted to PRP. Patients who were treated with PRP and did not have baseline DME had, on average, no change in RNFL thickness after one year of follow-up and an average decrease of less than 5 µm after 2 years (28). These data confirm that PRP, as performed in this study or in protocol S, has minor effects on RNFL thickness changes after one year. Patients receiving anti-VEGF injections had a different response than patients treated with PRP in protocol S, with an average loss of more than 10 µm in RNFL thickness after 2 years. The possible interference of DME and intravitreal anti-VEGF on RNFL thickness parameters led us to restrict our eligibility criteria for PDR patients without clinically significant macular edema. Moreover, two eyes that developed significant DME during the follow-up period were excluded from the analysis and were treated according to the current best clinical practices with anti-VEGF intravitreal injections.

A limitation of our study is the relatively small number of patients recruited. However, our sample is similar to other prospective interventional studies (7), and we adopted rigid inclusion criteria that excluded patients with optic disk neovascularization and/or baseline DME to achieve good internal validation. The presence of disk neovascularization does not allow an adequate analysis of the anatomy of the optic disc and peripapillary region by the GDx and OCT because fibrovascular tissue can obscure the edges of the optic cup and impair measurements of RNFL thickness (7). Moreover, DME is frequently present when PDR is detected in diabetic patients and often requires additional treatment with intravitreal anti-VEGF injections, which could somehow interfere with the parameters analyzed in our study. For proper documentation of the RNFL and to avoid the bias of anti-VEGF injections, only PDR cases without disc neovascularization and without macular edema were eligible for this particular study. We know from the diabetic retinopathy study (DRS) that only 40% of PDR cases do not present with disc neovascularization (2). The DRCR net protocol S (27), a multicentric and randomized clinical trial that compared PRP or anti-VEGF for PDR cases, reported that approximately 22% of participants with PDR had DME at baseline. Therefore, most PDR patients who presented at our center were not eligible for this study.

In conclusion, we prospectively followed PDR patients submitted to laser PRP. Data from our study show that PRP did not cause significant changes in RNFL peripapillary thickness after 1 year of treatment. Therefore, moderate intensity laser, as employed in this trial, can be used in PDR patients without causing significant changes in RNFL thickness, thus avoiding potential diagnostic confusion with glaucoma or its progression in such a population.

## AUTHOR CONTRIBUTIONS

Zacharias LC, Azevedo BMS and Araújo RB were responsible for the study conception and design, acquisition of data, analysis and interpretation of data, drafting of manuscript and critical revision. Ciongoli MR was responsible for the data acquisition, analysis and interpretation. Hatanaka M was responsible for the study conception and design, data analysis and interpretation, and manuscript critical revision. Preti RC was responsible for the manuscript critical revision. Monteiro MLR was responsible for the study conception and design, manuscript drafting and critical revision.

## Figures and Tables

**Table 1:** Comparison of scanning laser polarimetry (SLP) with variable corneal compensation (GDx VCC) parameters before and after panretinal photocoagulation (n=38).

Parameters	Pretreatment		Posttreatment		*p* *
	Mean±SD	Range	Mean±SD	Range	
TSNIT Avg (μm)	47.9±5.7	46.0-49.7	47.7±4.9	46.0-49.3	0.952
Superior Avg (μm)	59.5±8.5	56.7-62.3	58.8±7.4	56.3-61.2	0.464
Inferior Avg (μm)	58.4±9.3	55.3-61.4	58.9±8.8	56.0-61.8	0.541
TSNIT SD (μm)	22.4±3.8	21.2-23.7	22.4±4.0	21.1-23.8	0.743
NFI	26.9±10.1	23.5-30.2	28.4±10.5	25.0-31.9	0.354

SD, standard deviation; TSNIT, temporal-superior-nasal-inferior-temporal; NFI, nerve fiber indicator. Avg, Average.

* Wilcoxon signed rank-test.

**Table 2:** Average retinal nerve fiber layer (RNFL) thickness measures by spectral-domain optical coherence tomography before and 1 year after panretinal photocoagulation (n=38).

RNFL Thickness	Pretreatment	Posttreatment	*p* *
	Mean (μm)±SD	Mean (μm)±SD	
Global	97.2±13.4	96.0±12.3	0.469
Nasal Superior	106.9±23.2	105.0±19.8	0.410
Temporal Superior	133.3±20.6	132.0±18.9	0.413
Temporal	75.4±12.6	74.3±12.6	0.565
Temporal Inferior	140.1±24.1	139.0±22.9	0.168
Nasal Inferior	108.0±26.8	104.2±21.7	0.931
Nasal	70.0±12.8	68.9±15.3	0.121

RNFL, retinal nerve fiber layer; SD, standard deviation.

* Wilcoxon signed rank-test.
